# Rudolf Virchow

**DOI:** 10.3201/eid1409.086672

**Published:** 2008-09

**Authors:** Myron Schultz

**Affiliations:** National Center for Environmental Health/Agency for Toxic Substances and Disease Registry, Atlanta, Georgia, USA

**Keywords:** pathology, cell pathology, pathologic anatomy, Rudolf Virchow, photo quiz

**Figure:**
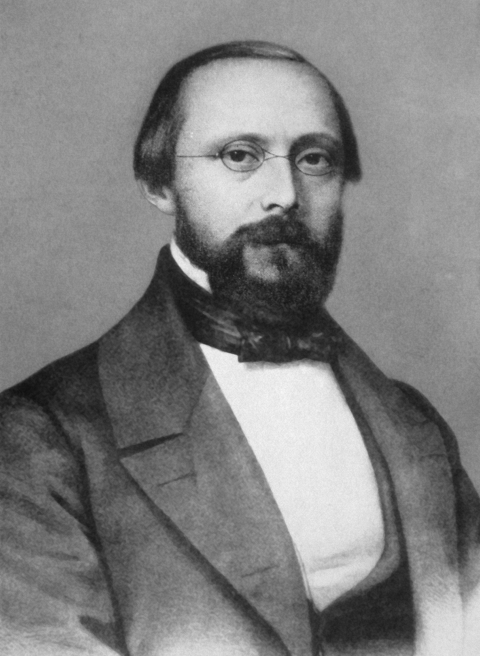


## Rudolph Virchow (1821–1902)

Virchow was one of the 19th century’s foremost leaders in medicine and pathology. He was also a public health activist, social reformer, politician, and anthropologist.

Virchow was the only child of a farmer and city treasurer in Schivelbein, Germany. He had a strong interest in natural science. In 1839, he received a scholarship from the Prussian Military Academy, where he was given the opportunity to study medicine in preparation for a career as an army physician. He studied medicine in Berlin and then taught there for the most of his life, with interludes in Silesia and Würzberg. In 1847, he and a colleague, Benino Reinhardt, founded the Archiv für Pathologische Anatomie und Physiologie (now known as “Virchow’s Archives”), which still survives as a leading journal of pathology. He encouraged his students to use microscopes and “think microscopically.” Virchow had a major impact on medical education in Germany. He taught several persons who became famous scientists in Germany, including Edwin Klebs, Ernst Haekel, and Adolf Kussmaul. He also taught William Welch and William Osler, 2 of the 4 famous physicians who founded Johns Hopkins Hospital.

Virchow’s greatest accomplishment was his observation that a whole organism does not get sick—only certain cells or groups of cells. In 1855, at the age of 34, he published his now famous aphorism “*omnis cellula e cellula*” (“every cell stems from another cell”). With this approach Virchow launched the field of cellular pathology. He stated that all diseases involve changes in normal cells, that is, all pathology ultimately is cellular pathology. This insight led to major progress in the practice of medicine. It meant that disease entities could be defined much more sharply. Diseases could be characterized not merely by a group of clinical symptoms but by typical anatomic changes. Pathologic anatomy, in addition to its great scientific merit, had tremendous practical consequences. If the physician was able to find out what anatomic changes had occurred in a patient, he could make a much more accurate diagnosis of the disease than he could in the past. This also empowered physicians to give more precise treatment and prognosis. In many of his speeches Virchow stated that the practice of medicine in Germany should shift away from being a largely theoretical activity. He advocated for the study of microscopic pathological anatomy, for research to be performed by physicians, the importance of making systematic clinical observations, and the performance of animal experimentations.

Virchow’s many discoveries include finding cells in bone and connective tissue and describing substances such as myelin. He was the first person to recognize leukemia. He was also the first person to explain the mechanism of pulmonary thromboembolism. He documented that blood clots in the pulmonary artery can originate from venous thrombi. While Virchow, in Germany, was developing the new science of cellular pathology, Louis Pasteur, in France, was developing the new science of bacteriology. Virchow fought the germ theory of Pasteur. He believed that a diseased tissue was caused by a breakdown of order within cells and not from an invasion of a foreign organism. We know today that Virchow and Pasteur were both correct in their theories on the causality of disease.

Virchow noted the link between diseases of humans and animals and coined the term “zoonosis” to indicate the infectious diseases links between animal and human health. In addition to his groundbreaking work in cellular pathology he created the field of comparative pathology. Yet, Virchow’s concept of “One Medicine,” was not uniformly appreciated during his lifetime.

In 1848, Virchow served on a commission to investigate an epidemic of typhus, for which he wrote a penetrating report that criticized the social conditions that fostered the spread of the disease. He had already established a reputation as a crusading social reformer, and this report consolidated that reputation. He has since been identified as much with what came to be called “social medicine” as with his primary specialty of pathology.

Virchow was an outspoken advocate for public health. His writings and teachings are full of observations and recommendations about ways to improve people’s health by improving their economic and social conditions. He entered politics, serving in the German Reichstag (1880–1893), while also directing the Pathological Institute in Berlin. He helped to shape the healthcare reforms introduced in Germany during the administration of Otto von Bismarck. His prolific writings, while mainly on topics of pathology, included many essays and addresses on social medicine and public health.

Among Virchow’s many interests was helminthology. He described the life cycle of *Trichinella spiralis* in swine and its zoonotic consequences. He was opposed to Bismarck’s excessive military budget, which angered Bismarck sufficiently to challenge Virchow to a duel. Virchow, being entitled to choose the weapons, chose 2 pork sausages: a cooked sausage for himself and an uncooked one, loaded with *Trichinella* larvae, for Bismark. Bismarck, the Iron Chancellor, declined the proposition as too risky.

Virchow also contributed substantially to anthropology, paleontology, and archeology. It should be noted that even men of great accomplishment, like Virchow, are fallible. Virchow believed that the Neanderthal man was a modern *Homo sapiens*, whose deformations were caused by rickets in childhood and arthritis later in life, with the flattened skull due to powerful blows to the head. Subsequent discoveries and research showed that the Neanderthals are, indeed, ancient.
